# Importance of integrating biological sex and age analyses in health research

**DOI:** 10.1186/s13293-026-00900-1

**Published:** 2026-05-12

**Authors:** DeLisa Fairweather, Georgios Kararigas

**Affiliations:** 1 Department of Cardiovascular Medicine, Mayo Clinic, 4500 San Pablo Road, Jacksonville, FL 32224 USA; 2 Department of General Internal Medicine, Mayo Clinic, 4500 San Pablo Road, Jacksonville, FL USA; 3 Center for Clinical and Translational Science, Mayo Clinic, 200 First Street SW, Rochester, MN USA; 4 Department of Basic and Clinical Sciences, Medical School, University of Nicosia, UNIC Athens, Athens, Greece

## Abstract

Research findings in human, animal and cell populations may be influenced by biological sex and age. The traditional statistical approach to sex and/or age differences that are discovered in research data is to consider them as confounders that interfere with the ability to make accurate estimations and thus need to be controlled for. This article provides examples of how combining sexes or controlling for sex and/or age may lead to inaccurate findings and overlook important insights. Rather than treating these variables as confounding factors, they should be viewed as variables of importance to the research question. We contend that data should be analyzed according to sex/age as the primary analysis, while controlling for sex and age should be conducted as a secondary analysis. This topic is increasingly important as data are made publicly available and deep learning models/artificial intelligence are used to analyze large volumes of data. Mechanistic insights will continue to be lost if prompt action is not taken to report data according to sex and age and to analyze data by sex and age in health research.

## Introduction

Research findings may be influenced by biological sex and age in human, animal and cell populations. Biological sex (i.e., cis-male vs. cis-female) is not only a demographic label but far more than that, sex differences exist in physiologic parameters, metabolism, and inflammatory responses, for example, in healthy and diseased individuals [1–6]. Similarly, age affects physiology, cognition, disease risk, mortality, immune function, and metabolism, among others [3, 7, 8]. These fundamental biological sex and age differences are thus expected to affect research data.

Many published manuscripts continue to *omit* the sex of the people, animals or cells in their study. Additionally, many studies do not *analyse* their data according to sex. For example, studies that use ANOVAs as the statistical approach may not include age/sex as factors- thus ignoring the effects of sex and/or age. A traditional statistical approach to prevent confounding in research is to *control* for sex and age. The net effect of controlling for sex and age is to ‘remove’ or ignore these factors in research instead of considering them as important factors that are critical for understanding health and disease in preclinical and clinical research. The goal of this article is to highlight how sex and age differences in data need to be analyzed rather than simply ‘controlled for’ and that this change in research and statistical perspective is necessary for breakthroughs in understanding health and disease, especially with the advent of artificial intelligence-based analysis of existing datasets.

## Controlling for sex and age

Sex and age are controlled for several reasons. In clinical and translational research, we often seek to determine how a risk factor affects a disease or disease outcome. A *confounder* can be defined as a variable (i.e., sex, age) that distorts the estimated association between a predictor and an outcome, because it is associated with the predictor and/or the outcome. Thus, if the confounder is not controlled for, there may be a biased estimate of the predictor-outcome relationship [9]. It is assumed that the confounder, sex and/or age, is not critical to the outcome (i.e., disease pathogenesis) of the condition being examined. However, if we are engaged in human or animal research, then sex and age are fundamentally involved in biological processes.

Every time we control for sex and/or age, we are treating these variables as confounding factors rather than variables of importance to the research question. [10] This approach assumes that the change in the variable being researched (i.e., predictor) is the same for men and women. By controlling for sex and/or age, we are ignoring a major part of the analysis of the data in preclinical and clinical research. And this is not only making the conclusions of research inaccurate, but also it may lead to potentially dangerous outcomes for patients. As described by Shapiro et al., an original study of HIV medications controlled for sex, but did not report sex differences in outcomes. [10] However, a later reanalysis of the data by sex found that women had higher adverse events and treatment discontinuation than men, which affected medication adherence. [10–12] This is just one example of many. Thus, if the effects of sex are controlled for rather than analyzed, study findings may not generalize to both sexes with important implications for patient care. Thus, we contend that data should be analyzed according to sex/age as the primary analysis, while controlling for sex and age should be conducted as a secondary analysis.

Ignoring the importance of the effects of sex can also lead to flawed inference if males and females are analyzed together. For example, soluble ST2 (sST2) has been discovered as a blood biomarker for heart failure for several cardiovascular diseases and for this reason it has been added fairly recently to the clinical panel of biomarkers used to assess risk of death from heart failure. [13] If male and female data are combined, sST2 appears to be a good biomarker for poor outcome in both sexes in patients with myocarditis comparing New York Heart Association (NYHA) heart failure grade with serum sST2 levels (*r* = 0.248, *p* < 0.0001) [14, 15]. However, if the data are examined by sex, elevated sST2 levels only correlate with worse cardiac outcome in males with myocarditis (*r* = 0.292, *p* < 0.0001), with no significant relationship found in females (*r* = 0.179, *p* = 0.08) [14, 15]. This study provides an example of how one sex may carry the finding in the data, which is absent in the other sex. Because sex differences for many biological factors go in opposite directions (i.e., high in one sex and low in the other), ignoring sex effects by combining males with females may prevent important discoveries, because combined data may inaccurately indicate that no relationship exists between the predictor and outcome.

### Sex and age are interrelated

Several articles have recently reviewed evidence for how sex and age, separately, affect biology and immune responses in health and disease [2, 4, 6]. Here, we want to highlight how sex and age are interrelated. Fundamentally, changes in gonadal hormone levels with age - from puberty to menopause in women and the steady decline of testosterone with age in men - illustrate how the two factors are integrated [3, 8]. Because gonadal hormones, in particular, influence the function of all cells in the body, in health and disease, transitions that occur in their levels and ratios over time influence every human and animal condition. These differences are reflected in peak occurrence or prevalence of diseases, such as autoimmune diseases in females at certain ages [8, 16]. Other examples include how asthma peaks in males prior to puberty but only after puberty in females, [17] higher incidence of strokes and death in men in early adulthood that occur in women later in life, [8] sex and age differences in blood pressure in patients with hypertension, [3] myocarditis peaks in young males around 35 years of age but cases mainly appear in women following menopause, [18] and hypermobile Ehlers-Danlos syndrome (hEDS) peaks in females around 35 years of age [19]. Following on with the earlier example of elevated blood sST2 levels predicting heart failure in myocarditis; not only did elevated sST2 levels only predict worse outcome in males with myocarditis, but it predicted worse outcome only in young males with myocarditis under 50 years of age [14]. These differences point to a sex-age interaction that influences the mechanism of disease, yet sex and age are routinely ignored in the analysis or controlled for. The effects of sex and age should no longer be ignored, but they must urgently be analyzed in order to better understand the pathogenesis of disease and to identify efficacious therapies.

## How can we improve?

First, we need to disaggregate data by sex and age to understand how they interact with each other and/or with the predictors and outcomes under investigation. This will reveal the true relationship between a predictor and outcome. This approach is extremely important as raw data are now required to be made publicly available for studies using US government funding and as the capacity to rapidly analyze large amounts of data is now available. The ability of artificial intelligence deep machine learning models to analyze data is only as good as the quality of the data available to the system [20]. In order for data to be analyzed correctly using these models, we need to ensure that all data provided on public repositories are disaggregated by sex and age. Insights will continue to be lost if we do not act fast to report data according to sex and age and to analyze by sex and age in our basic and clinical research. Secondly, the fundamental differences we observe in biology related to sex and age should be viewed *positively*, rather than negatively, as an opportunity to develop personalized medicine approaches to health and disease for each sex and age group. This *shift* in viewpoint happens most effectively when we experience the ‘light bulb’ moment and realize the impact analyzing our own data by sex/age has on the outcomes and interpretation of the data.
